# Effect of Central Longitudinal Dipole Interactions on Chiral Liquid-Crystal Phases

**DOI:** 10.3390/ijms19092715

**Published:** 2018-09-11

**Authors:** Takuma Nozawa, Paul E. Brumby, Kenji Yasuoka

**Affiliations:** Department of Mechanical Engineering, Keio University, 3-14-1 Hiyoshi, Kohoku-ku, Yokohama 223-8522, Japan; tnozawa@keio.jp (T.N.); p.brumby@keio.jp (P.E.B.)

**Keywords:** molecular simulation, liquid crystals, phase transition, chirality, dipole

## Abstract

Monte Carlo simulations of chiral liquid-crystals, represented by a simple coarse-grained chiral Gay–Berne model, were performed to investigate the effect of central longitudinal dipole interactions on phase behavior. A systematic analysis of the structural properties and phase behavior of both achiral and chiral systems, with dipole interactions, reveals differing effects; strong dipole interactions enhance the formation of layered structures; however, chiral interactions may prevent the formation of such phases under certain conditions. We also observed a short-ranged smectic structure within the cholesteric phases with strong dipole interactions. This constitutes possible evidence of presmectic ordering and/or the existence of chiral line liquid phases, which have previously been observed in X-ray experiments to occur between the smectic twisted grain boundary and cholesteric phases. These results provide a systematic understanding of how the phase behavior of chiral liquid-crystals changes when alterations are made to the strength of dipole interactions.

## 1. Introduction

The different types of thermotropic liquid-crystals can be characterized by varying degrees of molecular orientational and positional order. One of the most common is the nematic liquid-crystal phase. In the nematic phase, there is only orientational order but no positional order between molecules. The molecules of the smectic phase, on the other hand, are positioned such that they form a layered structure, in addition to their orientational order. There are many kinds of smectic phases, which differ in the particular arrangement of molecules within the layers.

**Figure 1:**
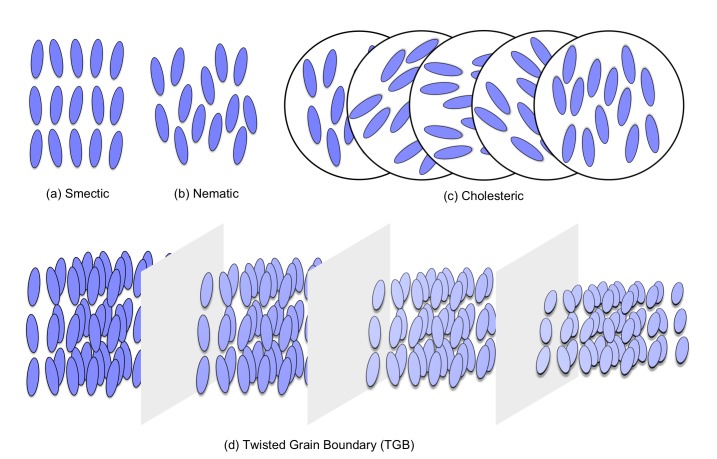
Schematic view of representative liquid-crystal phases. (**a**) smectic phase; (**b**) nematic phase; (**c**) cholesteric phase and (**d**) twisted grain boundary (TGB) phase.

Chirality is an important molecular characteristic that, when present, often results in formation of additional classes of liquid-crystal phases with interesting and useful optical properties. Under certain conditions, rod-like chiral molecules or molecular systems with a chiral dopant may arrange themselves into twisted super-structures. An example of this is the chiral-nematic phase, which has a continuously twisting helical structure. This phase is also referred to as the cholesteric phase, since it was first observed for cholesterol derivatives. Analogous to the relationship between nematic and cholesteric phases, the smectic twisted grain boundary (TGB) phase retains strong smectic ordering on a local scale, while exhibiting a larger-scale chiral rotation of the ordering director. In this case, the rotation occurs between adjacent slabs of smectic ordering, which are separated by grain boundaries. These boundaries are regularly spaced and this permits rotation of the director between slabs. Representative diagrams of these phases are shown in Figure 1. Further to these phases, the chiral line liquid phase has been observed from high-resolution calorimetry and X-ray experiments [1,2,3,4]. The chiral line liquid phase exists between the TGB and cholesteric phases. Previous works have reported that it has a short-range smectic layer formation, like those found in the TGB phases [2,4]. Following these previous works, several studies have been performed to investigate the phase behavior of the chiral line liquid, but these are few in number in comparison with other liquid-crystal phases [5,6,7,8,9,10,11]. The recent development of photonic technologies has enabled the creation tunable nanostructures of these phases. A variety of bulk phase properties are thus easily controllable. Examples include optically-tunable helical twisting power [12,13] and reflections [14], and light-driven handedness of helical superstructures inversion [15,16] as well as other-types of structural changes [13,17,18]. These developments will serve to promote the design and application of intelligent advanced functional materials [19].

The observed macroscopic structures of liquid crystals are strongly influenced by the collective behavior of their constituent molecules. Therefore, the study of these fluids at a molecular scale is important to further understanding of the origin of their bulk-scale physical properties. To study physics at a molecular scale, it is useful to carry out Monte Carlo or Molecular Dynamics simulations. These methods allow a better understanding of complex fluids, such as liquid crystals, at a microscopic scale, and reveal how molecular interactions give rise to the bulk scale phenomena commonly observed in experiments. For example, a recent simulation study on triangular prisms demonstrated how a chiral phase may form from molecules with purely steric interactions [20]. This finding partially supports experimental observations of helical filaments [21] and gives weight to the notion of a purely entropic mechanism behind the formation of some chiral nematic phases. Of course, the Gay–Berne model [22] and the hybrid Gay–Berne model [23,24,25,26,27] are useful to study the general behavior of liquid crystals, and are still commonly used. In part, this is because of their computational efficiency, but it is also due to the ease with which they may be tuned to allow focused examination of the effects of varying different molecular characteristics. There are notable examples of similar works for chiral liquid-crystals in the literature: Memmer et al. developed a simple coarse-grained model for chiral liquid-crystals, and demonstrated their phase behavior [28,29,30,31,32,33,34]; Allen et al. investigated the structure of the TGB phase by introducing modified boundary conditions [35]; Berardi et al. studied chiral interactions of a nematic phase with a chiral surface [36]; thermomechanical coupling and heat conduction in a cholesteric phase were studied by Sarman et al. [37]; Melle and Schlotthauer et al. studied nano-confined systems of chiral liquid-crystals [38,39]; and chiral superstructures were investigated using a linear rigid coarse-grained model by Yan et al. [40]. The studies mentioned above employed simple models, but give a general understanding of the characteristics of chiral liquid-crystals. As such, they are useful to predict physical properties of fluids of real molecules which share similar characteristics.

Dipole interactions are also an important characteristic to consider for liquid crystals, since they promote quick response times to the imposition of electric fields, while dipolar liquid-crystal materials are often used for industrial products. For achiral liquid crystals with dipoles, several studies have been reported. For example, we know from a number of Monte Carlo simulations of dipolar hard-spherocylinders that the addition of central dipoles, be they longitudinal or transverse, stabilizes the smectic-*A* phase at the expense of the nematic phase [41,42,43]. Conversely, terminal longitudinal dipoles increase the stability of the nematic phase while reducing that of the smectic-*A* phase [42,43,44,45]. An earlier study by Gwóźdź et al. [46] reported that the Gay–Berne system with central transverse dipoles does not yield an appreciable difference in the phase stability of the smectic phase, relative to the non-polar system, but does enhance the ordering within it.

As noted above, many studies have been conducted to promote a better understanding of chiral liquid crystals at the molecular scale. There are, to date, no theoretical or simulation studies that combine both chiral and dipolar electrostatics. In this study, therefore, we carefully investigate the effect of central longitudinal dipole interactions on the structure of chiral liquid crystals using Monte Carlo simulations. The current paper will serve to provide the first such analysis of the interplay between dipolar and chiral electrostatics and their effect on the phase behavior of liquid crystal fluids. The subsequent sections of this paper are arranged as follows: in Section 2, the results of these simulations are presented; the conclusions drawn from these findings are made in Section 3, and the methods used in our simulations are detailed in Section 4.

## 2. Results and Discussion

### 2.1. The Effect of Chirality on Non-Polar Liquid Crystals

**Figure A1:**
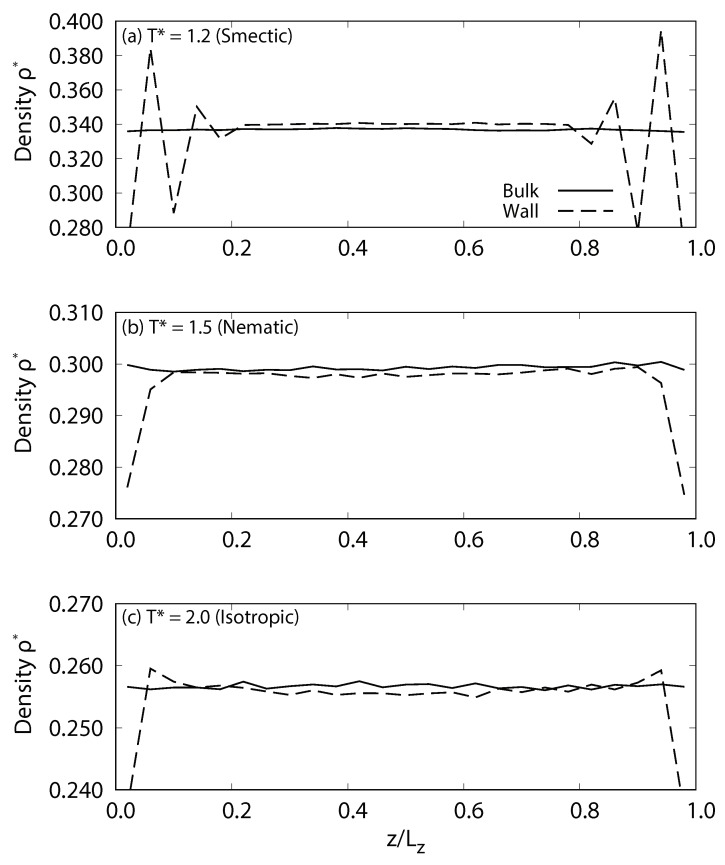
Local density profiles for achiral liquid-crystal systems (c=0.0) in different phases: smectic ($T^{*}=1.2$), nematic ($T^{*}=1.5$), and isotropic ($T^{*}=2.0$). The continuous lines correspond to bulk systems, while the dashed lines represent confined systems in the presence of solid substrates.

**Figure A2:**
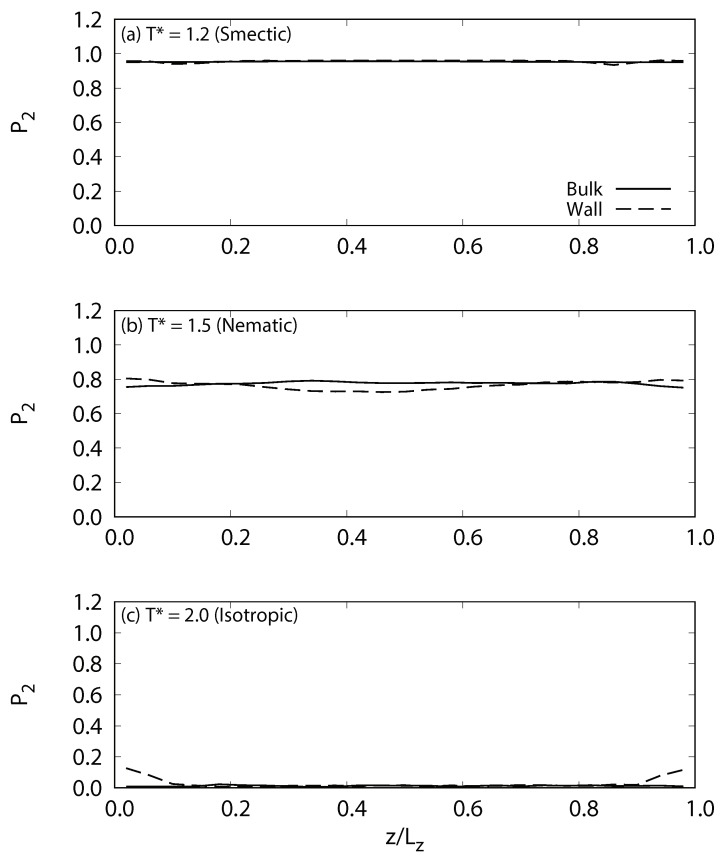
Local profiles of orientational order parameter $P_{2}$ for achiral liquid-crystal systems (c=0.0) in different phases: smectic ($T^{*}=1.2$), nematic ($T^{*}=1.5$), and isotropic ($T^{*}=2.0$). The continuous lines correspond to bulk systems, and the dashed lines represent confined systems in the presence of solid substrates.

Temperature-dependent phase transitions for non-polar, chiral, liquid crystals were extensively studied by Memmer et al.; however, their simulations were performed under full 3D-periodic boundary conditions that may have induced serious artifacts in the chiral phase due to unrealistic deformation of the equilibrium pitch. Therefore, we performed MC simulations for non-polar liquid crystals at similar conditions with planar walls to investigate the phase behavior carefully. All averaged physical properties for confined systems are calculated excluding molecules near the substrates because those molecules are strongly affected by the repulsive interactions and anchoring effects of the surface, as shown in Figure A1 and Figure A2. The simulation domain is divided into slices of equal volume, with each stacked on top of a neighbouring slice, in the direction of the *z*-axis. All physical properties are calculated separately for each slice, and averaged over all slices from $z/L_{z}$ = 0.2 to 0.8.

**Figure 2:**
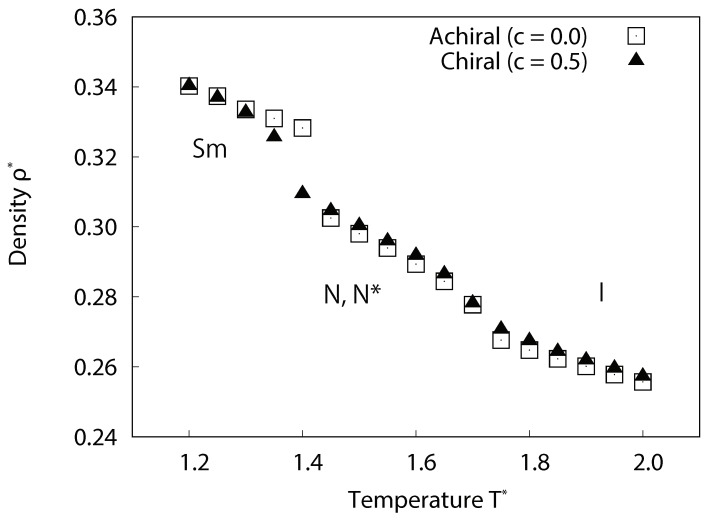
Temperature dependence of the averaged local density for achiral and chiral liquid-crystal systems without dipoles. For achiral systems (c=0.0), smectic phases occur at lower temperature, nematic phases at the intermediate temperature range, and isotropic phases at higher temperature. For chiral systems (c=0.5), similarly, smectic phases occur at lower temperature, cholesteric phases at the intermediate temperature range, and isotropic phases at higher temperature. The system densities of chiral phases are generally higher than those of achiral phases.

**Figure 3:**
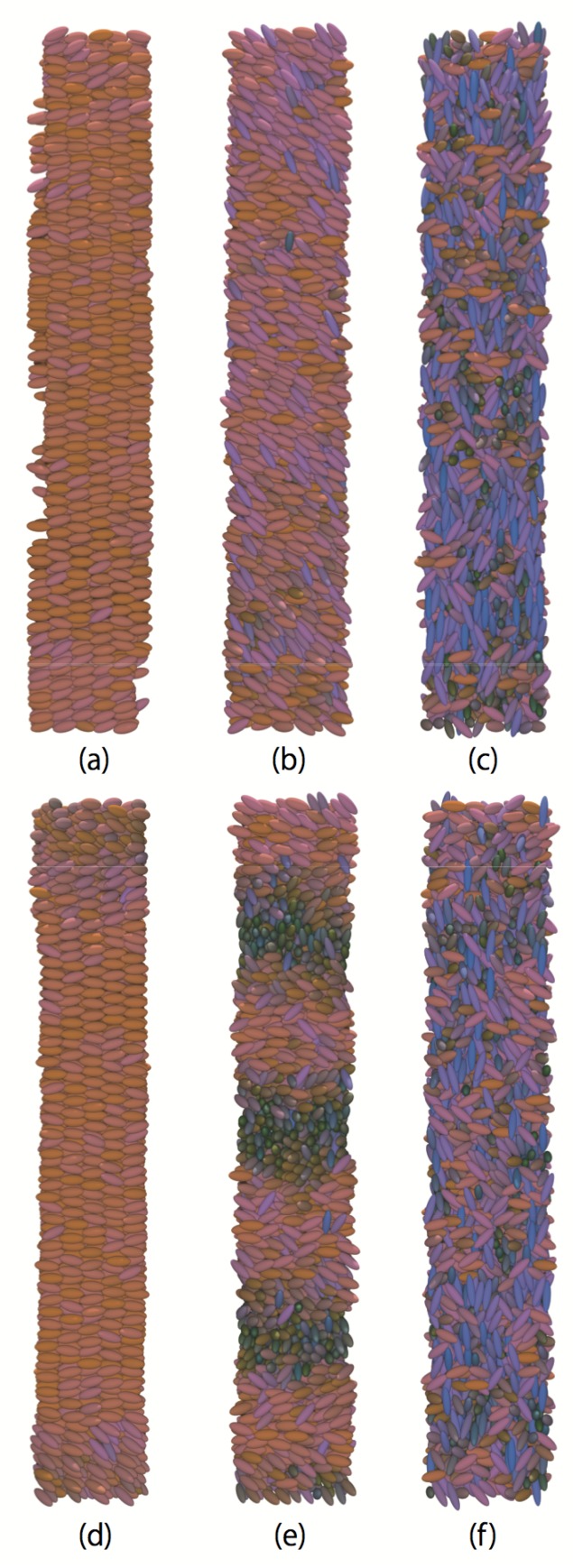
Representative snapshots of the non-polar achiral system (c=0.0): (**a**) smectic phase, at lower temperature ($T^{*}=1.2$); (**b**) nematic phase at the intermediate temperature ($T^{*}=1.5$); (**c**) isotropic phase at higher temperature ($T^{*}=2.0$), and the non-polar chiral system (c=0.5): (**d**) smectic phase, at lower temperature ($T^{*}=1.2$); (**e**) cholesteric phase at the intermediate temperature ($T^{*}=1.5$); and (**f**) isotropic phase at higher temperature ($T^{*}=2.0$).

**Figure 4:**
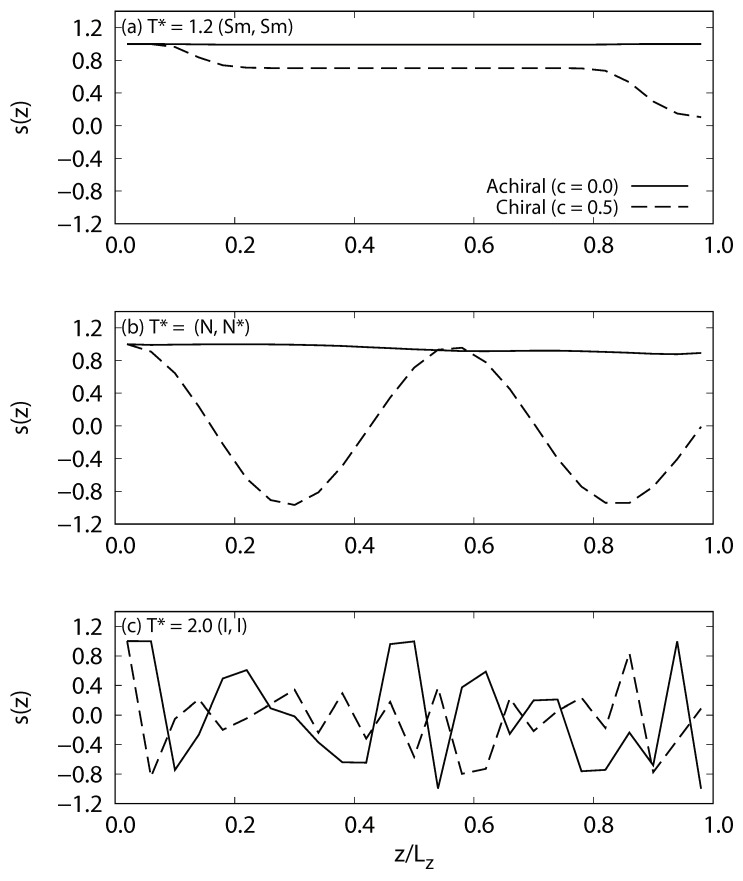
Local profiles of the director correlation function in the *z*-axis $s\left(z\right)=\langle n_{0}\cdot n_{z}\rangle$ for achiral and chiral liquid-crystal systems without dipoles, where $n_{z}$ is the local orientation director along the *z*-axis. For achiral systems (c=0.0), strong correlations of director are observed for smectic (Sm) and nematic (*N*) phases; they are ordered in one direction. For chiral systems (c=0.5), director correlation changes near the solid substrates for smectic (Sm) because of the wetting effect. The director correlation of cholesteric phase ($N^{*}$) decays because of the helical structure. For the isotropic phases (*I*) in the achiral and chiral systems, random director correlations are a characteristic of this phase.

To examine the effect of chirality on phase behavior and system density, the temperature dependence of the averaged local density, as shown in Figure 2, is examined. First order phase transitions can be characterized by a sudden and sharp change in density relative to the change in temperature. The density decreases with temperature and two changes in gradient were observed, which implies the existence of three distinct phases for both achiral and chiral systems (c=0.0 and 0.5). Examination of the snapshots (Figure 3) and director profiles (Figure 4) reveals their identity. For achiral systems (c=0.0), smectic phases occur at low temperature, nematic phases at the intermediate temperature range, and isotropic phases at high temperature. The smectic-nematic phase transition occurs at $1.4\leq T^{*}\leq 1.45$, and the nematic-isotropic phase transitions occur at $1.7\leq T^{*}\leq 1.75$. For chiral systems (c=0.5), isotropic phases are present at high temperatures, while, in the intermediate temperature range, we see cholesteric phases. At the lowest temperatures, we also observe smectic phases. In fact, it is difficult to distinguish TGB phases from smectic phases in our studies due to limitation of systems size and wetting effects by solid substrates. The director correlation profile for *z*-axis s(z) decays as shown in Figure 4 and twisted structures may appear when the system is enough large; however, we here denote these phases as smectic tentatively. The smectic-cholesteric transition occurs at temperatures of $1.35\leq T^{*}\leq 1.4$, and the cholesteric-isotropic phase transition occurs at $1.7\leq T^{*}\leq 1.75$. Interestingly, the density of chiral systems are generally higher than those of the achiral, non-smectic, phases at the same temperatures. This increased density may be caused by the additive chiral potential $\propto -{\left(1/r\right)}^{7}$, which may act to reduce molecule-to-molecule separation distances and potential well depth (see Appendix B). One should note this phenomenon is not found only for chiral Gay–Berne fluids, but also for other additive-type pair potentials, which are often used to model chiral interactions.

**Figure 5:**
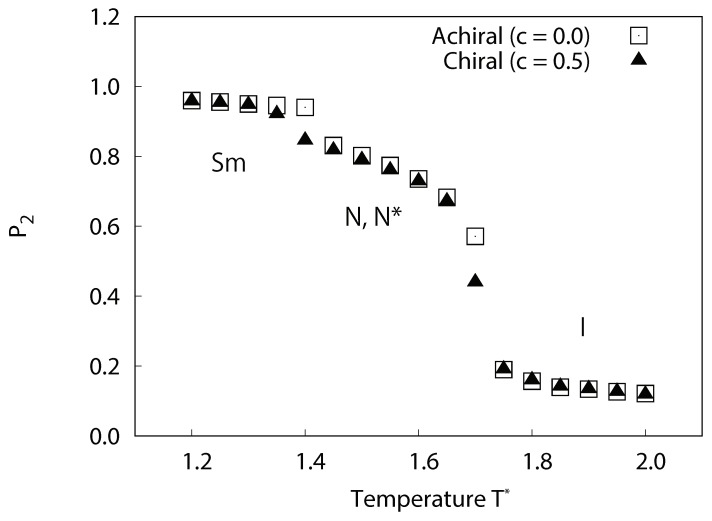
Temperature dependence of the averaged local orientational order parameter $P_{2}$ for achiral and chiral liquid-crystal systems without dipoles. For achiral systems (c=0.0), smectic phases occur at a lower temperature, nematic phases at the intermediate temperature range, and isotropic phases at higher temperature. For chiral systems (c=0.5), similarly, smectic phases occur at a lower temperature, cholesteric phases at the intermediate temperature range, and isotropic phases at higher temperature. The local orientational order of chiral systems is almost equivalent to those of the achiral systems.

The structural properties of our simulated systems were examined by comparison of their orientational ordering. In Figure 5, the temperature dependences of the orientational order parameter for each of the simulated systems are plotted. For the achiral systems (c=0.0), slight decreases in $P_{2}$ were observed at $1.4\leq T^{*}\leq 1.45$, which corresponds to the smectic-nematic phase transition. Significant decreases in $P_{2}$ were observed at $1.65\leq T^{*}\leq 1.75$ which corresponded to nematic-isotropic phase transitions. For chiral system (c=0.5), similarly, slight decreases in $P_{2}$ were observed at $1.35\leq T^{*}\leq 1.4$ corresponding to the smectic-cholesteric phase transition. Significant decreases in $P_{2}$ were observed at $1.65\leq T^{*}\leq 1.75$ which corresponded to cholesteric-isotropic phase transitions. Figure 5 clearly indicates that local orientational order in the chiral systems is almost equivalent to those found in the bulk of the achiral systems.

**Figure 6:**
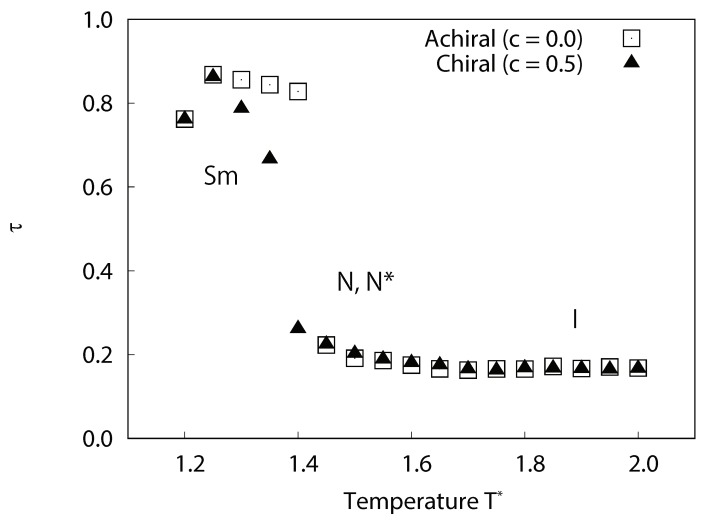
Temperature dependence of the averaged local translational order parameter τ for achiral and chiral liquid-crystal systems without dipoles. For achiral systems (c=0.0), smectic phases occur at a lower temperature, nematic phases at the intermediate temperature range, and isotropic phases at a higher temperature. For chiral systems (c=0.5), similarly, smectic phases occur at lower temperature range, cholesteric phases at the intermediate temperature range, and isotropic phases at higher temperature. This figure reveals clear evidence of layered structures found in smectic phases.

In the second stage of our structural analysis, the translational order parameter of the equilibrium systems was calculated using Equation (9). The temperature dependence of translational order parameter τ are plotted in Figure 6. For achiral system (c=0.0), significant decreases in τ at $1.4\leq T^{*}\leq 1.45$ corresponded to smectic-nematic phase transitions. For chiral system (c=0.5), significant decreases in τ at $1.35\leq T^{*}\leq 1.4$ corresponded to smectic-cholesteric phase transitions.

**Figure 7:**
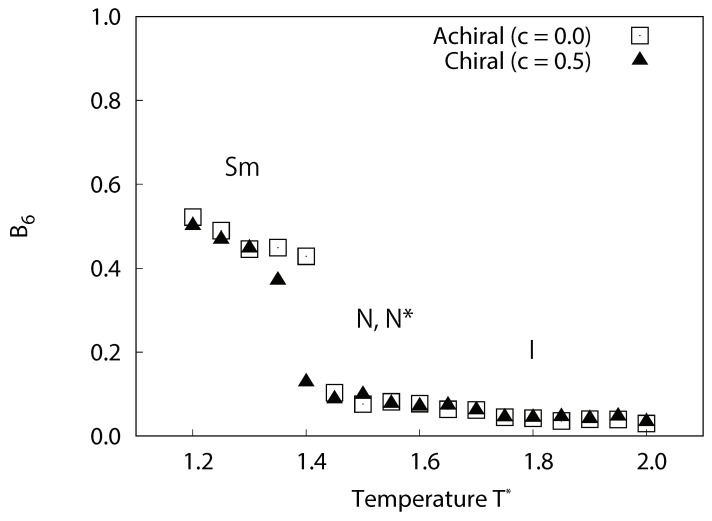
Temperature dependence of the averaged local six-fold bond order parameter $B_{6}$ for achiral and chiral liquid-crystal systems without dipoles. For achiral systems (c=0.0), smectic phases occur at lower temperature, nematic phases at the intermediate temperature range, and isotropic phases at higher temperature. For chiral systems (c=0.5), similarly, smectic phases occur at lower temperature, cholesteric phases at the intermediate temperature range, and isotropic phases at higher temperature. The figure clearly indicates that the smectic phases obtained from our simulations have a local hexagonal packing structure, which has similarities to that of the smectic-*B* phase.

In the third stage of our structural analysis, the bond order parameter of the equilibrium systems was calculated using Equation (10). In Figure 7, the temperature dependences of the six-fold bond order parameter $B_{6}$ for each system are plotted. For achiral system (c=0.0), significant decreases in $B_{6}$ at $1.4\leq T^{*}\leq 1.45$ corresponded to smectic-nematic phase transitions. For chiral system (c=0.5), significant decreases in $B_{6}$ at $1.35\leq T^{*}\leq 1.4$ corresponded to smectic-cholesteric phase transitions. This indicates that the smectic phases obtained from our simulations have a local hexagonal packing structure, which has similarities to that of the smectic-*B* phase.

### 2.2. Effect of Dipole Interactions

In this section, the effect of central longitudinal dipole interactions on the structural properties of chiral liquid crystals was studied. At low temperatures, we observed the occurrence of metastable structures. For this reason, our focus here is restricted to a slightly higher temperature range: $1.5\leq T^{*}\leq 2.0$.

**Figure 8:**
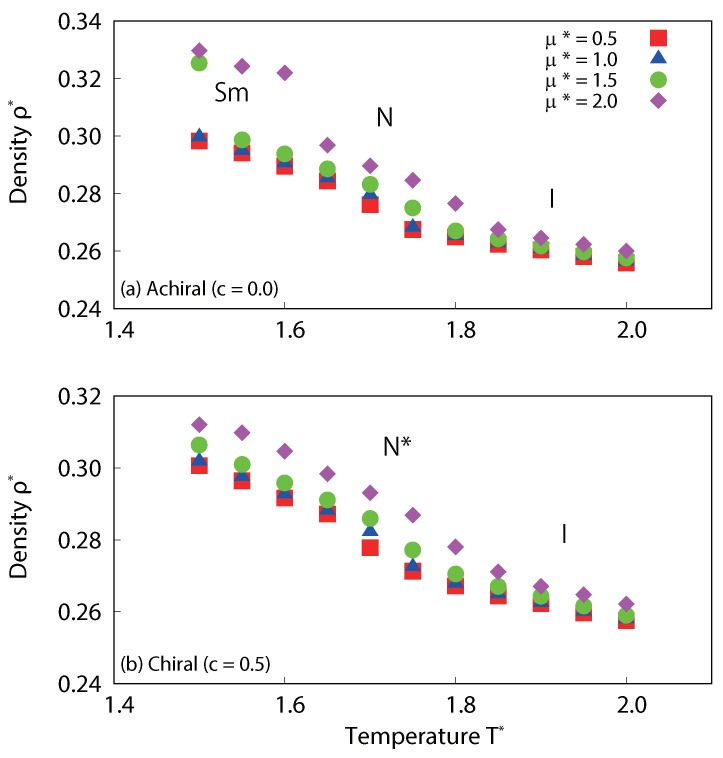
Temperature dependence of the averaged local density for (**a**) achiral (c=0.0) and (**b**) chiral (c=0.5) liquid-crystal systems with dipole interaction. For achiral systems, nematic and isotropic phases occur for $\mu^{*}=0.5$ and 1.0, and smectic phases occur in addition to these two phases for $\mu^{*}=1.5$ and 2.0. For chiral systems, on the other hand, cholesteric and isotropic phases occur for all dipole conditions. For both achiral and chiral systems, density increases with dipole strength.

We now examine how the phase behavior and system density are effected by the addition of dipoles. As with the non-polar systems above, this is done by inspection of the temperature dependence of the averaged local density, which is displayed in Figure 8. For achiral systems (c=0.0), two phases were observed in the temperature range; nematic phases occur at $1.5\leq T^{*}\leq 1.65$, and isotropic phases at $1.75\leq T^{*}\leq 2.0$ for weak dipoles ($\mu^{*}=0.5$ and 1.0), similar to our results for non-polar systems. For strong dipoles ($\mu^{*}=1.5$ and 2.0), three phases were observed; smectic phases occur at low temperature, nematic phases at the intermediate temperature range, and isotropic phases at high temperature. The results clearly indicate that the smectic-nematic and nematic-isotropic phase transition points are shifted to higher temperatures with increased dipole strength. This tendency is consistent with previous work by Satoh et al. [47] and by Houssa et al. [48]. For chiral systems (c=0.5), on the other hand, only two phases were observed for the four dipole strengths investigated ($\mu^{*}=0.5$, 1.0, 1.5 and 2.0); cholesteric phases occur at low temperature, and isotropic phases at high temperature. This highlights a difference in how the addition of dipole interactions affects achiral and chiral systems. Normally, the central longitudinal dipole interactions enhance the formation of layered structures, as demonstrated by the enhanced stability of the smectic phase with increasing dipole strength in the achiral systems. In the case of the chiral systems, however, the chiral interactions suppress the formation of such layered structures. In Figure 8, we see clearly that the system density increases with dipole strength; however, the effect is not significant for isotropic phase, relative to the ordered phases. In the isotropic phase, molecular behavior is almost entirely random and thus we do not see the creation of that stable dipolar domains which appear in ordered phases. It is for this reason that the density of the isotropic phase is less sensitive to the presence or absence of dipoles.

**Figure 9:**
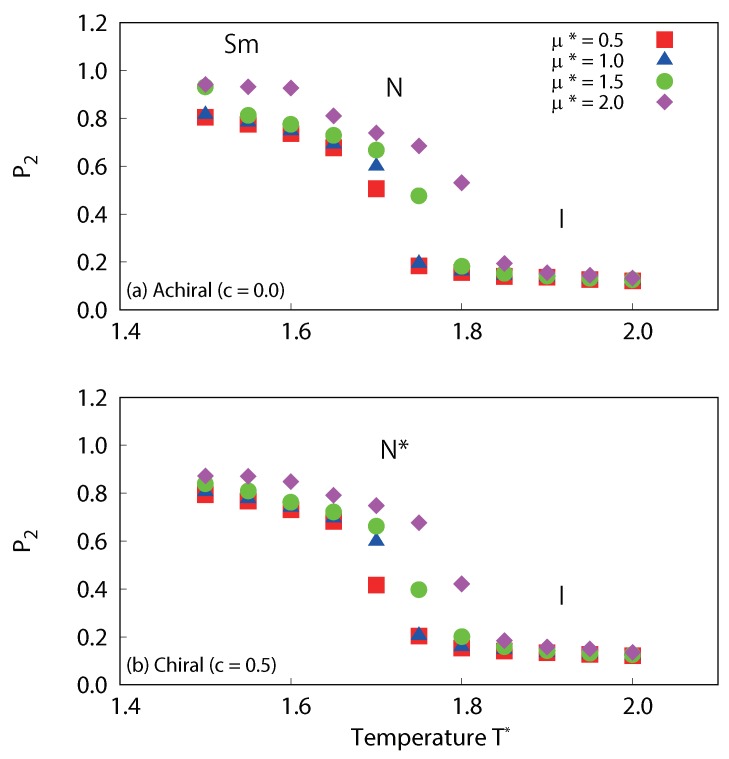
Temperature dependence of the averaged local orientational order parameter $P_{2}$ for (**a**) achiral (c=0.0) and (**b**) chiral (c=0.5) liquid-crystal systems with dipole interactions. Slight decreases in $P_{2}$ are observed, which correspond to smectic-nematic phase transitions of achiral system, and significant decreases in $P_{2}$ are observed, which corresponds to nematic-isotropic transition of an achiral system or cholesteric-isotropic phase transition of a chiral system.

The structural properties of our simulated systems were examined by comparison of their orientational, translational and bond ordering. In Figure 9, the temperature dependences of the orientational order parameter for dipolar liquid crystals are plotted. For the achiral system (c=0.0), significant decreases in $P_{2}$ were observed, which corresponded to nematic-isotropic phase transitions for all dipole strengths ($\mu^{*}=0.5$, 1.0, 1.5 and 2.0). For strong dipole interactions ($\mu^{*}=1.5$ and 2.0), slight decreases in $P_{2}$ were also observed, which correspond to smectic-nematic phase transitions. For the chiral system (c=0.5), significant decreases in $P_{2}$ were observed which corresponded to cholesteric-isotropic phase transitions, for all dipole strengths ($\mu^{*}=0.5$, 1.0, 1.5 and 2.0).

**Figure 10:**
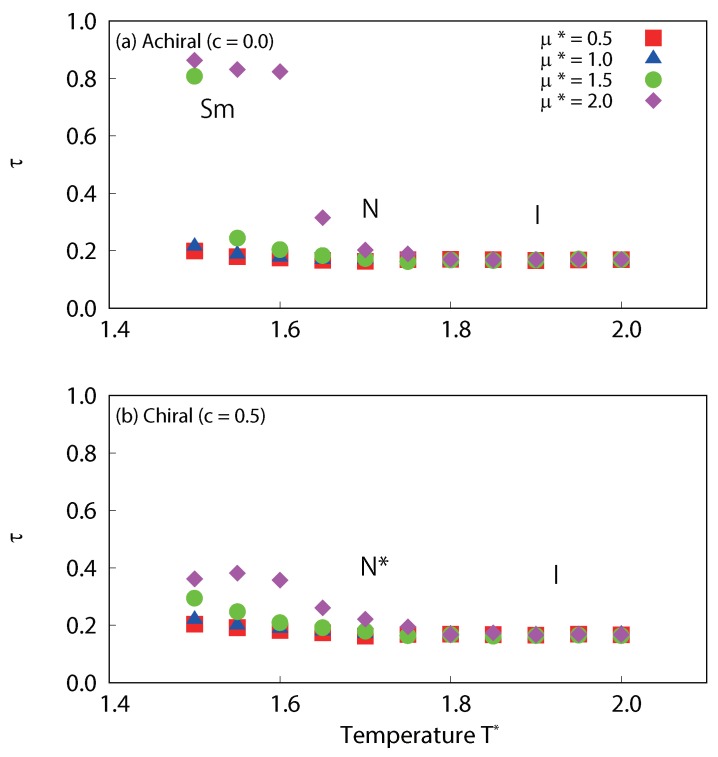
Temperature dependence of the averaged translational order parameter τ for (**a**) achiral (c=0.0) and (**b**) chiral (c=0.5) liquid-crystal systems with dipole interactions. Significant decreases in τ are observed, which correspond to smectic-nematic phase transitions of achiral systems. The figure clearly indicates that τ increases with dipole strength, especially for nematic and cholesteric at low temperature. τ for cholesteric phases with $\mu^{*}=2.0$ at $1.5\leq T^{*}\leq 1.6$ is appreciably larger than others.

**Figure 11:**
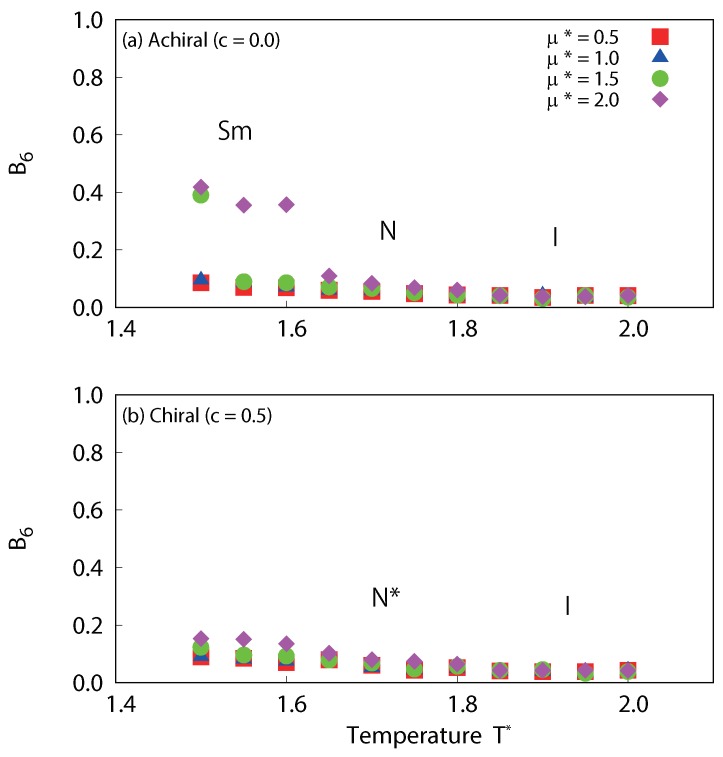
Temperature dependence of the averaged local six-fold bond order parameter $B_{6}$ for (**a**) achiral (c=0.0) and (**b**) chiral (c=0.5) liquid-crystal systems with dipole interactions. Significant decreases in $B_{6}$ are observed, which correspond to smectic-nematic phase transitions of achiral systems.

**Figure 12:**
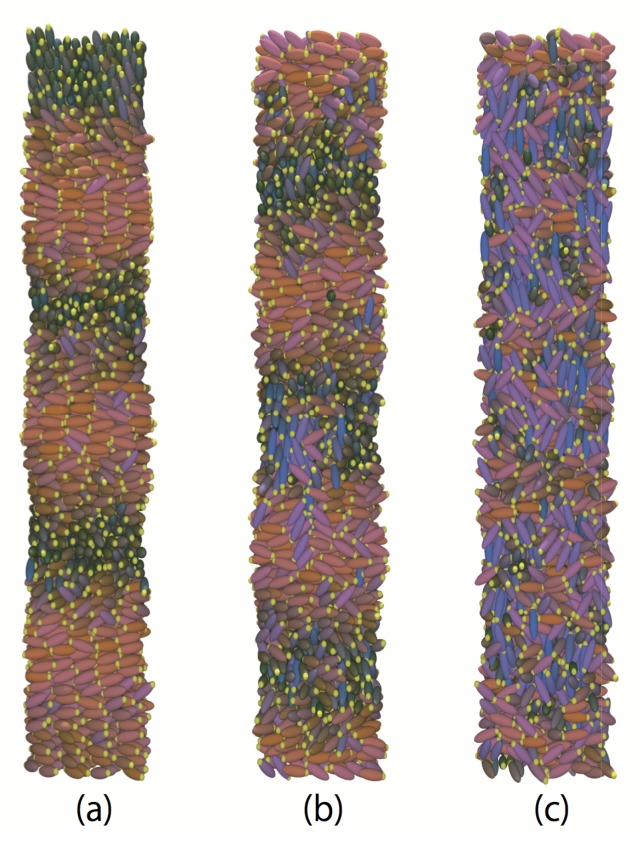
Representative snapshots of the chiral (c=0.5) with $\mu^{*}=2.0$: (**a**) cholesteric phase with short-range smectic layer at $T^{*}=1.5$; (**b**) cholesteric phase at $T^{*}=1.7$, and (**c**) isotropic phase at $T^{*}=2.0$. The yellow head shows dipole direction.

The temperature dependences of the translational order parameter τ are plotted in Figure 10. For the achiral system (c=0.0) with strong dipole interactions ($\mu^{*}=1.5$ and 2.0), sharp decreases in τ are observed at low temperature. This corresponds to smectic-nematic phase transitions. On the other hand, the values of τ for chiral systems at low temperature are much smaller than those of achiral systems. Interestingly, τ increases with dipole strength both for achiral and chiral systems. This clearly indicates that dipole interactions promote layered structures; however, the transition to a full smectic phase is inhibited when chiral interactions are present. Moreover, τ for cholesteric phases with $\mu^{*}=2.0$ at $1.5\leq T^{*}\leq 1.6$ is appreciably larger than the other cases, a clear sign of possible presmectic ordering. Presmectic ordering [49] in liquid crystals, which may occur at high fluid densities, just below the nematic to smectic phase transition point, have been observed in several experiments [50,51]. The existence of short-range presmectic peaks in structure factor profiles was first predicted theoretically [52], and then subsequently observed in simulation [53]. In Figure 11, the temperature dependences of the six-fold bond order parameter $B_{6}$ for dipolar liquid crystals are plotted. In agreement with the study by Houssa et al. [54], we note that smectic-*A* phases were not obtained in our studies, either with or without a central dipole. For the achiral system (c=0.0), there are significant decreases in $B_{6}$ at low temperature for strong dipole conditions ($\mu^{*}=1.5$ and 2.0), which correspond to smectic-nematic phase transitions. On the other hand, there is no such change for the chiral system (c=0.5). This also indicates that there is a difference in the structures obtained at low temperature between the achiral and chiral systems. Examination of the snapshots (Figure 12) reveals differences in τ of cholesteric phases. The cholesteric phases for $\mu^{*}=2.0$ at $1.5\leq T^{*}\leq 1.6$ appears to have short-range smectic ordering as shown in Figure 12a. These phases appear to exhibit regions of presmetic ordering, and/or the characteristics of the chiral line liquid phase, but further investigation will be required to identify this phase precisely. One should also note that there may be hysteresis for the smectic-nematic and nematic-isotropic phase transitions. According to previous studies [55,56], this hysteresis will be negligibly small, at least for non-polar Gay–Berne systems (see Figures 4 and 5 of [55] and Figure 2 [56]). Even with dipolar interactions, hysteresis at the nematic-isotropic phase transition has not been observed [48]. However, some hysteresis may occur at the smectic-nematic phase transition. A significant hysteresis has been observed for a mixture of Lennard-Jones and Gay–Berne models at strong charges or high density conditions (see Figures 2 and 7 of [57]), thus hysteresis may be increased with these cases. Further investigation will be required to determine the precise locations of the phase transition points between smectic-nematic or smectic-cholesteric phases.

## 3. Conclusions

To design highly functional liquid-crystal devices, a clear and systematic understanding of how molecular characteristics influence the structure and phase behavior is necessary. In this study, molecular simulations for model liquid-crystals were performed to investigate the effect of chirality and dipole strength upon the phase behavior. Using a series of analysis methods, accurate phase identities and phase transitions were studied in a systematic manner. Interestingly, the effect of introducing dipole interactions on structure and phase behavior is different for achiral and chiral systems. For achiral systems, dipole interactions promote liquid-crystal ordering, and smectic-nematic and nematic-isotropic phase transition points are shifted to higher temperatures with increased dipole strength. For chiral systems, on the other hand, the transition to a smectic phase does not occur in the temperature range investigated, although the cholesteric-isotropic phase transition is shifted to higher temperatures, as with an achiral system. The chiral interactions inhibit the formation of smectic phases. Moreover, strong dipoles may induce a short-range presmectic ordering in cholesteric phases at low temperature, and may result in a phase that is structurally similar to the chiral line liquid phase. Future work will of course be required to confirm this hypothesis.

For the chiral systems, the focus of our study was restricted to the cholesteric and isotropic phases since many metastable structures were observed at lower temperatures. The sampling of TGB-like phases by standard MC simulation is challenging; however, an extended ensemble approach such as multi-canonical simulation [58] or the generalized replica exchange method [59] may overcome this problem. Furthermore, these methods will also address the issue of hysteresis between smectic and nematic phases. System size is also a factor that limits the scope of our investigations. If found, the TGB phase will have a local smectic structure with a very long pitch distance. Large-scale simulation of such systems may be required to obtain reliable equilibrium data for the TGB phase, which presents a considerable computational burden.

## 4. Methods

### 4.1. Fluid–Fluid Interactions

In this work, we make use of a modified version of the Gay–Berne (GB) potential, developed by Memmer et al. [32]. This potential allows for the simulation of chiral liquid-crystal fluids and includes additive chiral and dipole interaction terms. The full potential is

(1) $$U_{ff}\left({\hat{u}}_{i},{\hat{u}}_{j},r_{ij}\right)=U_{\mathrm{GB}}\left({\hat{u}}_{i},{\hat{u}}_{j},r_{ij}\right)+cU_{c}\left({\hat{u}}_{i},{\hat{u}}_{j},r_{ij}\right)+U_{d}\left({\hat{u}}_{i},{\hat{u}}_{j},r_{ij}\right),$$

where ${\hat{u}}_{i}$ and ${\hat{u}}_{j}$ are the orientation vectors of molecules *i* and *j*, respectively, $r_{ij}$ is the unit vector of the inter-molecular center-to-center vector $r_{ij}=r_{j}-r_{i}$, and *c* is the chiral strength parameter. $U_{\mathrm{GB}}\left({\hat{u}}_{i},{\hat{u}}_{j},r_{ij}\right)$ is the standard GB potential:

(2) $$U_{\mathrm{GB}}\left({\hat{u}}_{i},{\hat{u}}_{j},r_{ij}\right)=4\epsilon \left({\hat{u}}_{i},{\hat{u}}_{j},{\hat{r}}_{ij}\right)\left\{{\left(\frac{\sigma_{0}}{r_{ij}-\sigma \left({\hat{u}}_{i},{\hat{u}}_{j},{\hat{r}}_{ij}\right)+\sigma_{0}}\right)}^{12}-{\left(\frac{\sigma_{0}}{r_{ij}-\sigma \left({\hat{u}}_{i},{\hat{u}}_{j},{\hat{r}}_{ij}\right)+\sigma_{0}}\right)}^{6}\right\}.$$

The chiral term, $U_{c}\left({\hat{u}}_{i},{\hat{u}}_{j},r_{ij}\right)$, is given by

(3) $$U_{c}\left({\hat{u}}_{i},{\hat{u}}_{j},r_{ij}\right)=-4\epsilon \left({\hat{u}}_{i},{\hat{u}}_{j},{\hat{r}}_{ij}\right)\left\{{\left(\frac{\sigma_{0}}{r_{ij}-\sigma \left({\hat{u}}_{i},{\hat{u}}_{j},{\hat{r}}_{ij}\right)+\sigma_{0}}\right)}^{7}\left[\left({\hat{u}}_{i}\times {\hat{u}}_{j}\right)\cdot {\hat{r}}_{ij}\right]\left({\hat{u}}_{i}\cdot {\hat{u}}_{j}\right)\right\}.$$

It should be noted that the parameters σ and ϵ in Equation (3) are identical to those of the standard GB potential.

The dipole term, $U_{d}\left({\hat{u}}_{i},{\hat{u}}_{j},r_{ij}\right)$ is given by

(4) $$U_{d}\left({\hat{u}}_{i},{\hat{u}}_{j},r_{ij}\right)=\frac{\mu_{i}\mu_{j}}{r_{ij}^{3}}\left\{{\hat{u}}_{i}\cdot {\hat{u}}_{j}-3\left({\hat{u}}_{i}\cdot {\hat{r}}_{ij}\right)\left({\hat{u}}_{j}\cdot {\hat{r}}_{ij}\right)\right\},$$

where ${\hat{\mu }}_{i}$ and ${\hat{\mu }}_{j}$ are dipole moments of molecules *i* and *j*, namely, ${\hat{\mu }}_{i}$ = $\mu_{i}{\hat{u}}_{i}$ and ${\hat{\mu }}_{j}$ = $\mu_{j}{\hat{u}}_{j}$ where $\mu_{i}$ and $\mu_{j}$ are the dipole strengths.

### 4.2. Fluid–Substrate Interactions

Three-dimensional periodic boundary conditions are often used in such simulations to represent a bulk state, but they sometimes induce serious artifacts in chiral phases due to inappropriate periodic images, unless the box length along the twisted axis is an integer of half the pitch distance. In this work, therefore, we used solid substrates with planar anchoring [38,60,61] along the axis of helical rotation in order to break symmetry and eliminate boundary artifacts in that axis. The fluid-substrate potential energy is expressed as

(5) $$\begin{array}{cc} U_{fs}\left({\hat{u}}_{i},z_{i}\right) & =U_{fs}^{\left(1\right)}\left({\hat{u}}_{i},z_{i}\right)+U_{fs}^{\left(2\right)}\left({\hat{u}}_{i},z_{i}\right)\\ & =\epsilon_{fs}\{a_{1}{\left(\frac{\sigma_{0}}{z_{i}}\right)}^{10}-a_{2}\frac{\exp(-\eta z_{i})}{z_{i}}g\left({\hat{u}}_{i}\right)\\ & +a_{1}{\left(\frac{\sigma_{0}}{L_{z}-z_{i}}\right)}^{10}-a_{2}\frac{\exp(-\eta \left(L_{z}-z_{i}\right))}{L_{z}-z_{i}}g\left({\hat{u}}_{i}\right)\}, \end{array}$$

where $\epsilon_{fs}$ is the depth of the attractive well and $z_{i}$ is the perpendicular distance between molecule *i* and the lower substrate. $L_{z}$ is the cell length of the *z*-axis, thus $L_{z}-z_{i}$ is the perpendicular distance from the upper substrate to the center of molecule *i*. $g({\hat{u}}_{i})$ is the anchoring function, which depends on molecular orientation. In this study, we employed the following function:

(6) $$g\left({\hat{u}}_{i}\right)={\left({\hat{u}}_{i}\cdot {\hat{e}}_{x}\right)}^{2}+{\left({\hat{u}}_{i}\cdot {\hat{e}}_{y}\right)}^{2}.$$

Here, ${\hat{e}}_{x}$ and ${\hat{e}}_{y}$ are the unit vectors along the *x*-axis and the *y*-axis of the Cartesian coordinate system, respectively. The parameters $a_{1}$ and $a_{2}$ determine the location of the minimum of the fluid-substrate potential. They are unique functions of the screening length $\eta^{-1}$ and are given by

(7) $$\begin{array}{cc} a_{1} & =\frac{1+\eta \sigma }{9-\eta \sigma },\\ a_{2} & =\frac{10\exp(\eta \sigma )}{9-\eta \sigma }. \end{array}$$

### 4.3. Orientational Order Parameter

In order to perform orientational analysis for liquid-crystal phases, the second rank orientational order parameter, $P_{2}$, is often used. The order parameter $P_{2}$ is the largest eigenvalue of the ordering matrix Q, which takes the form of

(8) $$Q=\frac{1}{2N}\sum_{i=1}^{N}\left(3{\hat{u}}_{i}⊗{\hat{u}}_{i}-I\right),$$

where *N* is the number of molecules and I is the identity matrix. If all molecules in the system are oriented along the same axis, $P_{2}$ is equal to 1, while $P_{2}$ is equal to 0 if molecule oritentations are distributed randomly.

### 4.4. Translational Order Parameter

To identify the layered structures of smectic phases, the translational order parameter τ is commonly used. In this study, the following equation is used to compute τ:

(9) $$\tau \left(d\right)=∥\sum_{j=1}^{N}\exp\left(2\pi ir_{j,||}/d\right)∥,$$

where $r_{j,||}=r_{j}\cdot \hat{n}$ is the coordinate parallel to the director $\hat{n}$ and *d* is layer spacing. The translational order parameter τ and the layer spacing are given by the maximum value of τ(d).

### 4.5. Bond Order Parameter

Some liquid-crystal phases have positional order within layered structures, in addition to orientational order. To characterize such positional ordering, it is useful to consider the bond order parameter. This function is defined as

(10) $$B_{\nu }=\frac{1}{\nu N}∥\sum_{j=1}^{N}\sum_{k=1}^{\nu }\exp\left(i\nu \phi_{jk}\right)∥,$$

where $\phi_{jk}$ is the angle between bond-linked particles *j* and *k*, and a fixed reference frame. ν is the number of nearest-neighbor bonds. In this study, ν=6. This value is chosen in order to identify local hexagonal packing, of the type typically found in smectic-*B*-like phases.

### 4.6. Simulation Conditions

Monte Carlo simulations were performed for both achiral and chiral systems with 3456 liquid-crystal molecules. To represent these liquid-crystal molecules, we use the chiral Gay–Berne potential, with a chirality parameter of c=0.0 for achiral molecules, and c=0.5 for chiral molecules. The GB potential usually contains four parameters (κ, $\kappa^{\prime }$, μ, ν) which determine the anisotropy in the molecular interactions. Molecular shape anisotropy is quantified by $\kappa \equiv \sigma_{e}/\sigma_{s}$, where $\sigma_{e}$ and $\sigma_{s}$ are the molecule size parameters for end-to-end and side-by-side configurations, respectively. The amount of anisotropy is determined by μ, ν, and $\kappa^{\prime }\equiv \epsilon_{e}/\epsilon_{s}$. Here, $\epsilon_{e}$ and $\epsilon_{s}$ are the well depths for the end-to-end and side-to-side configurations, respectively. For our study on the effects of interaction-strength anisotropy, the potential parameters (κ, $\kappa^{\prime }$, μ, ν) = (3.0, 0.2, 1.0, 2.0) were used. These potentials are truncated using the spherical cutoff method, and potentials are shifted to reduce cutoff artifacts. In addition, solid substrates with planar anchoring are introduced in the *z*-axis, and 2D-periodic boundary conditions are used in the *x*- and *y*-axes to mimic the effect of large bulk systems while eliminating periodic boundary artifacts along the axis of helical rotation. In this work, all quantities are described using conventional reduced properties: reduced temperature $T^{*}=k_{B}T/\epsilon_{0}$; reduced pressure $P^{*}=P\sigma_{0}^{3}/\epsilon_{0}$; reduced potential energy per particle $U^{*}=U/\epsilon_{0}$; reduced distance $r^{*}=r/\sigma_{0}$; reduced density $\rho^{*}=N/V\sigma_{0}^{3}$, and reduced dipole strength $\mu^{*}=\mu /\sqrt{\sigma_{0}^{3}\epsilon_{0}}$. The cutoff radius for the potential was set at a reduced length of $r_{c}^{*}=\kappa +2.0=5.0$. All simulations were performed at a constant reduced pressure tangential to the substrates of $P_{xx}^{*}=P_{yy}^{*}=3.5$. Several isotherms in the range $T^{*}=1.2-2.5$ were investigated. In order to prevent unrealistic cell deformation, the cell length in the *z* direction is fixed, and anisotropic volume changes are attempted by rescaling the cell lengths in the other two cartesian axes. For the fluid–substrate interactions, $\eta^{-1}=\sigma_{0}$ and $\epsilon_{fs}=10\epsilon_{0}$ were chosen. The dipole strength $\mu^{*}$ was set to the following five values: 0.0, 0.5, 1.0, 1.5 and 2.0, in order to examine the relationship between the system’s phase behavior and the dipole moment. For the initial state of the dipolar systems, an isotropic configuration was used in order to reduce the chances of the systems becoming trapped at meta-stable local minima during the equilibration stage. If meta-stable states do occur during this process, those simulations are restarted from the most recent configurations of a neighboring temperature. For each state point, a long MC simulation was run for $4\times 10^{6}$ MC cycles to obtain equilibrium conditions. A further $1\times 10^{6}$ MC cycles were then performed to calculate equilibrium averages.

## Figures and Tables

**Figure A3:**
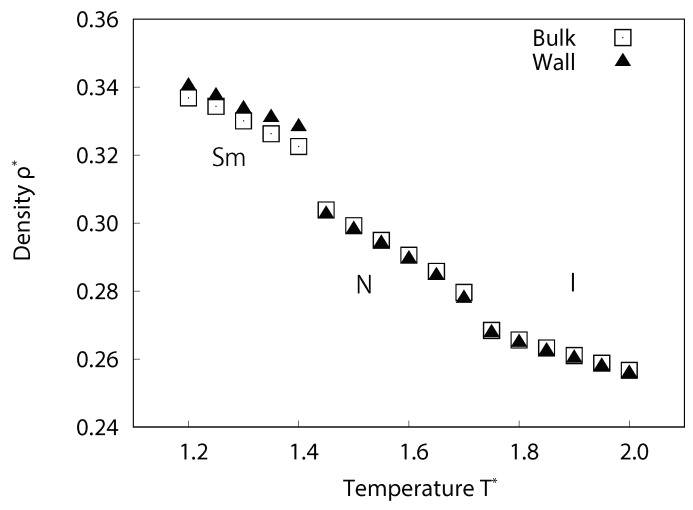
Temperature dependence of the averaged local density for bulk and confined liquid-crystal systems (c=0.0). The open squares correspond to bulk systems, and the filled triangles represent confined systems with the solid substrates. For both systems, smectic phases (Sm) occur at low temperature, nematic phases (*N*) at the intermediate temperature range, and isotropic phases (*I*) at high temperature.

**Figure A4:**
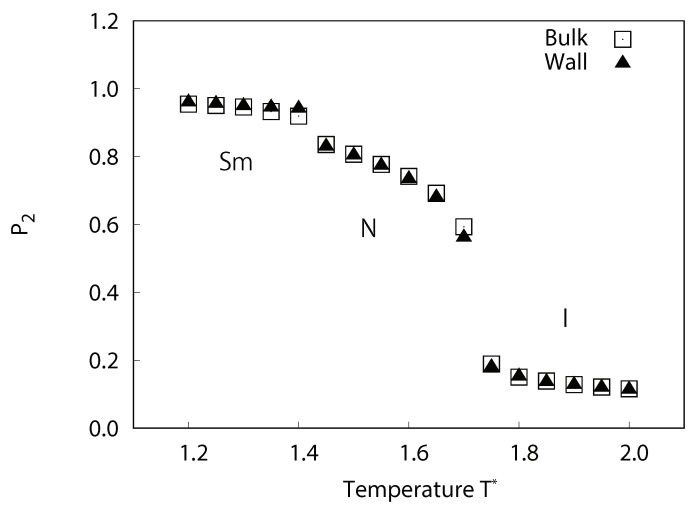
Temperature dependence of the averaged local orientational order parameter $P_{2}$ for bulk and confined liquid-crystal systems (c=0.0). The open squares correspond to bulk systems, and the filled triangles represent confined systems with the solid substrates. For both systems, smectic phases (Sm) occur at low temperature, nematic phases (*N*) at the intermediate temperature range, and isotropic phases (*I*) at high temperature.

**Figure A5:**
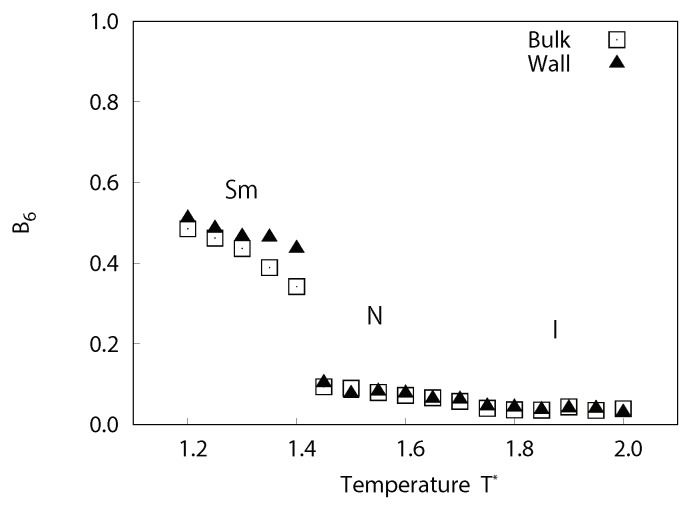
Temperature dependence of the averaged local six-fold bond order parameter $B_{6}$ for bulk and confined liquid-crystal systems (c=0.0). The open squares correspond to bulk systems, and the filled triangles represent confined systems with the solid substrates. For both systems, smectic phases (Sm) occur at low temperature, nematic phases (*N*) at the intermediate temperature range, and isotropic phases (*I*) at high temperature.

**Figure A6:**
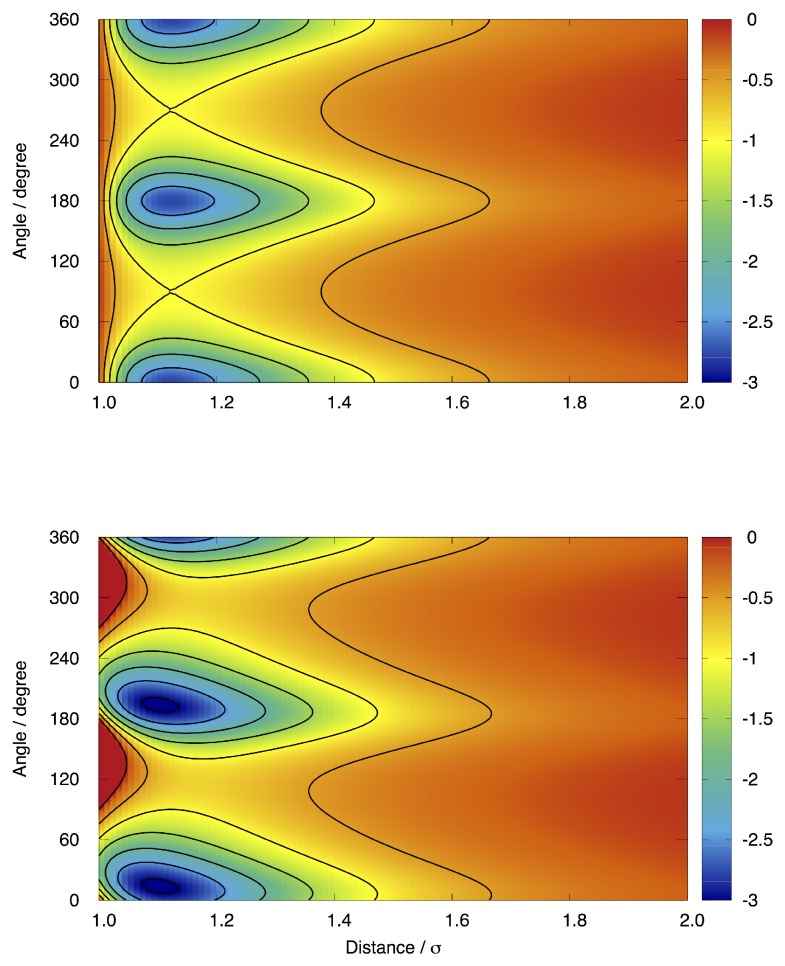
Energy contour maps: (**upper**) standard Gay–Berne potential with potential parameters (κ, $\kappa^{\prime }$, μ, ν) = (3.0, 0.2, 1.0, 2.0); (**below**) chiral Gay–Berne potential with potential parameters (κ, $\kappa^{\prime }$, μ, ν) = (3.0, 0.2, 1.0, 2.0) and c=0.5.

## Appendix A. Effect of Confinement by Solid Substrates

Confinement by solid substrates affects local structures, and may change the phase behavior of the system as a whole. In this section, therefore, the simulation results for bulk and confined systems were compared in order to investigate the effect of the substrate. Figure A1 and Figure A2 show local density profiles and local profiles of the orientational order parameter $P_{2}$ in the direction of the *z*-axis, respectively. Figure A1 shows that the density decreases near the substrates, regardless of the phase type, because of the repulsive interaction between fluid and substrate. For the smectic phase, the density in the central portion of the confined system is larger than that of the bulk system, but the density of the central region for nematic and isotropic phases under confinement is almost the same as their corresponding bulk phase densities. Figure A2 shows that orientational order increases in proximity to the substrates for nematic and isotropic phases, but this effect is not observed for the smectic phase. The lower substrate induces slightly more surface ordering than the upper substrate. This is due its directional anchoring effect.

For the next step, the temperature dependence of several properties were compared in order to investigate the effect of substrates on phase behavior. All averaged physical properties for confined systems are calculated excluding molecules near the substrates because they are strongly affected by the repulsive interactions and surface anchoring effects, as shown in Figure A1 and Figure A2. The simulation domain is divided into slices of equal volume, each stacked on top of a neighbouring slice, in the direction of the *z*-axis. All physical properties are calculated for each slice separately, and then averaged over slices from $z/L_{z}$ = 0.2 to 0.8, which corresponds to the bulk region. Figure A3, Figure A4 and Figure A5 show the temperature versus the averaged local density, the averaged local orietational order parameter $P_{2}$, and the averaged local six-fold bond order parameter $B_{6}$, respectively. In Figure A3, there are two significant decreases in the density, one at $1.4\leq T^{*}\leq 1.45$ and the other at $1.7\leq T^{*}\leq 1.75$ for both bulk and confined systems, which implies the existence of three distinct phases. For both systems, smectic phases occur at low temperature, nematic phases at the intermediate temperature range, and isotropic phases at high temperature. The density of smectic phases for confined systems are higher than those of the bulk system. On the other hand, the density for nematic and isotropic phases in the central region of the confined systems is almost the same as their corresponding bulk system densities. Figure A4 shows that there are significant decreases in $P_{2}$ at $1.4\leq T^{*}\leq 1.45$ and $1.7\leq T^{*}\leq 1.75$, which corresponded to smectic-nematic and nematic-isotropic phase transitions, respectively. Figure A5 shows that there is a significant decrease in $B_{6}$ at $1.4\leq T^{*}\leq 1.45$, which corresponds to the smectic-nematic phase transition. This clearly indicates that, for both systems, the smectic phase has a local hexagonal packing structure like that found in the smectic-*B* phase, but it is worth noting that the $B_{6}$ values are higher for the confined systems.

These results clearly indicate that liquid-crystal molecules near substrates are strongly affected by the repulsive interactions and surface anchoring effects. This may lead to the formation of locally ordered structures that are different from those found in the bulk phase. This is especially true for the smectic phase; however, the bulk phase behavior of these systems was not found to change with confinement. It is therefore possible to study the phase behavior of liquid-crystal systems, in the presence of confinement, which can be used to eliminate inappropriate periodic images which would otherwise be problematic in chiral systems.

## Appendix B. Chiral Gay–Berne potential

Energy contour maps are plotted in Figure A6 to help explain the reasons behind the density increases for chiral systems. The additive-type chiral potential has potential minima at a molecule-to-molecule separation distance of $r^{*}=0$, thus the separation distance and potential well depth of chiral Gay–Berne model decreases, relative to the achiral model. For c=0.5, the separation distance is reduced from $r^{*}=1.12$ to $r^{*}=1.1$, and the well depth from −2.777289 to −3.080405.
